# The Ant-like *Tachydromia* Complex in the Iberian Peninsula—Insights from Habitat Suitability Modelling for the Conservation of an Endemism (Diptera: Hybotidae)

**DOI:** 10.3390/insects12121068

**Published:** 2021-11-29

**Authors:** Ana Rita Gonçalves, Carlos Vila-Viçosa, João Gonçalves

**Affiliations:** 1 Programa de Pós-Graduação em Entomologia, Instituto Nacional de Pesquisas da Amazônia, Av. André Araújo, 2936, Petrópolis, CP 478, Manaus CEP 69011-970, Brazil; 2 CIBIO (Research Center in Biodiversity and Genetic Resources)—InBIO (Research Network in Biodiversity and Evolutionary Biology), University of Porto, Campus Agrário de Vairão, Rua Padre Armando Quintas, 4485-661 Vairão, Portugal; Cvv@cibio.up.pt (C.V.-V.); joaofgo@gmail.com (J.G.); 3 MHNC-UP—Museum of Natural History and Science of the University of Porto—PO Herbarium, University of Porto, Praça Gomes Teixeira, 4099-002 Porto, Portugal; 4 Biology Department, Faculty of Sciences, University of Porto, Rua do Campo Alegre, 4169-007 Porto, Portugal; 5 BIOPOLIS Program in Genomics, Biodiversity and Land Planning, CIBIO, Campus de Vairão, 4485-661 Vairão, Portugal; 6 proMetheus—Research Unit in Materials, Energy and Environment for Sustainability, Instituto Politécnico de Viana do Castelo (IPVC), Avenida do Atlântico, No. 644, 4900-348 Viana do Castelo, Portugal

**Keywords:** species distribution modelling, habitat suitability, native oak forests, flightless

## Abstract

**Simple Summary:**

The ant-like flies are a group of nine species of flightless *Tachydromia*, with their distribution restricted to the Iberian Peninsula. Severe knowledge gaps regarding their distribution and ecological requirements hinder conservation assessments. To improve this scenario, an ensemble of 9 different species distribution models was applied to unveil habitat suitability and to provide guidelines for future studies. The most important factors influencing habitat suitability are climate-related, followed by forest type and structure, with well-defined biogeographic gradients. *T. lusitanica* and *T. ebejeri* are adapted to the mild temperature and high-humidity environments, typical of the Temperate–Eurosiberian life zone. *T. semiaptera* and *T. iberica* are adapted to progressively drier and hotter central and southern parts of the Iberian Peninsula, connected to transitional Temperate–submediterranean areas. Ant-like flies’ distribution overlaps with deciduous/ marcescent oak species, which can effectively indicate their presence in Iberia. Additionally, southern marcescent forests emerge as “islands” with particular interest for future fieldwork. Ant-like flies are threatened by several factors such as climate change and habitat destruction, including urbanization and forest fires. This study provides vital tools to better assess the ant-like flies’ conservation status and to manage their habitat.

**Abstract:**

Ant-like flies comprise nine Iberian endemic species of flightless *Tachydromia*. Severe knowledge gaps on distribution and ecological requirements hinder conservation assessments. Species distribution models were applied to unveil habitat suitability and to provide guidelines for future studies. An ensemble modeling approach combining ten different techniques was implemented with the biomod2 package. Occurrence data was partitioned into six sets, including two multi-species groups and four species. The most relevant drivers of habitat suitability are climate-related, followed by forest type and structure, according to well-defined biogeographic gradients. *T. lusitanica* and *T. ebejeri* are adapted to mild temperatures and high-humidity environments. Their distribution is connected to the Temperate–Eurosiberian life zone. *T. semiaptera* and *T. iberica* are adapted to progressively drier and hotter central and southern parts of the Iberian Peninsula, connected to transitional Temperate–submediterranean areas. Ant-like fly’ ranges overlap with deciduous/marcescent oak species, acting as suitable indicators of their presence in Iberia. Southern marcescent forests emerge as “islands” with particular interest for future prospections. Ant-like flies are threatened by several factors such as climate change and habitat destruction, including urbanization and forest fires. This study provides vital tools to better assess the ant-like flies’ conservation status and to manage their habitat.

## 1. Introduction

The Iberian ant-like *Tachydromia* species group includes nine phylogenetic related taxa [1] with a restricted distribution across the Iberian Peninsula, which can be roughly separated from all other species within the same genus by their lack of flight ability due to having wings totally absent or highly reduced/ modified, including a complete absence of halteres. These species inhabit forest leaf litter, a dynamic and complex environment in which arthropods play a key role in several ecological processes, particularly as organic matter decomposers [2]. The role of predator macroinvertebrates in shaping the communities that occur in leaf litter is still poorly understood. However, predator-detritivore-detritus food chains are fundamental to ecosystem functioning, e.g., increasing nutrient cycling [3]. Conversely, the abiotic factors present in the leaf litter seem to be significant to determine habitat suitability for predators and their prey, including stable humidity and temperature conditions [4].

Soil-dwelling larvae of Diptera, such as those that inhabit leaf litter, are an essential and often the most abundant element of the edaphon in a wide range of ecosystems, yet they are inadequately known [5]. Soil Diptera tend to be more abundant and diverse in forests than in arable soils, with the families Sciaridae, Cecidomyiidae, and Phoridae being the predominant taxa in previously studied European terrestrial ecosystems [6]. In certain forest ecosystems of the Iberian Peninsula, Sciaridae and Chironomidae are heavily predated by the Iberian ant-like *Tachydromia* species Meigen, 1803 (Hybotidae), which are also leaf litter dwellers [1]. Adults of these species can be very abundant at the local scale and, due to their predatory nature, likely play an important role in several ecosystems. It is expected that the larvae are also predators and soil dwellers, as observed for related species [7].

*Tachydromia* species are often present in climactic deciduous and marcescent forests, primarily dominated by *Quercus* L. spp. and *Fagus sylvatica* L. [1], mainly across temperate bioclimatic belts, but also in transitional submediterranean areas [8,9,10]. Most populations are found in highly fragmented landscapes, often due to disturbances of anthropic origin, driven by land-use change and invasive plant species. Additionally, they are also present in landscapes bordering mature forests that are recovering after abandonment.

At lower altitudes, the adults become active from the end of January/early February until the end of May, while the populations occurring at higher altitudes (e.g., in the Pyrenees) become active from May-onwards [1]. The species seem to have relatively similar ecological requirements at a broad scale, and some species co-occur locally and/or temporally.

Occurrence patterns suggest a notable ecological affinity of the ant-like flies with the distinct biogeographic areas and Life zones of the Iberian Peninsula [10,11]. However, it is necessary to understand better how these species are distributed and the similarities in geographic and environmental characteristics of their niches. Such biogeographic insights are essential to aid conservation and biodiversity management, making it possible to understand and assess species distributions and uncover species responses to a plethora of different interactions with biotic and abiotic variables [12].

Nonetheless, there are critical gaps concerning the geographic distribution and the ecological requirements determining the habitat suitability of Iberian ant-like flies, with most species known only from a few locations. In invertebrates, this is a common scenario that often hinders the development of conservation plans [13]. However, Habitat Suitability Models (HSM) or Species Distribution Models (SDM) can be used as a guiding tool to overcome knowledge gaps and to manage focal species and their habitats [14].

By employing an ensemble modelling approach [15,16] which combines multiple HSMs based on different modelling algorithms, we aim to: (i) understand the potential distribution and habitat suitability for the Iberian ant-like flies by analyzing the environmental variables that shape the distribution of different taxa at local and global scales; (ii) analyze the relationship between taxa distribution and Iberian climax forest-types and Life zones, and (iii) provide general guidelines for species conservation, future prospection, and management.

## 2. Materials and Methods

### 2.1. Study Area

**Figure 1:**
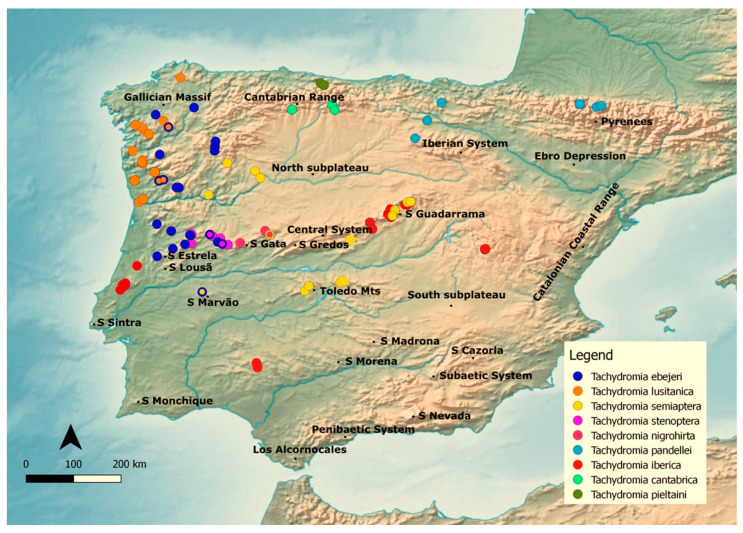
Topography of the Iberian Peninsula. The dots represent recorded species distribution. All localities mentioned in this work are indicated on the map. Adapted from Gonçalves et al., 2021.

The study area encompasses the Iberian Peninsula and includes the French portion of the Pyrenees (Figure 1), which is overall characterized by a complex orography encompassing two major biogeographic subregions: Eurosiberian and Mediterranean [10,17].

### 2.2. Target Taxa and Data Sources

The analyses are focused on the Iberian ant-like *Tachydromia*, which contains the following nine species: *Tachydromia iberica* (Arias, 1919), *Tachydromia semiaptera* (Gil Collado, 1923), *Tachydromia pieltaini* (Gil, 1936), *Tachydromia pandellei* (Séguy, 1941), *Tachydromia lusitanica* (Grootaert et al., 2009), *Tachydromia ebejeri* Gonçalves et al., 2021, *Tachydromia stenoptera* Gonçalves et al., 2021, *Tachydromia cantabrica* Gonçalves et al., 2021 and *Tachydromia nigrohirta* Gonçalves et al., 2021. Model development involved two distinct approaches: (i) pooling species data into two groups, the first one including all nine merged species dataset (hereafter, MSD) and, the second, aggregating records from species most phylogenetically related cluster (hereafter, PRC) and, (ii) by keeping species data individually thus resulting in a total of six different sets of data (Table 1).

Dataset 1 (MSD) merges all species’ records assuming that all nine Iberian ant-like *Tachydromia* have similar macro-climate requirements, allowing the analysis of the largest possible amount of presence records, which is generally reduced for each species or species subsets. Dataset 2 (PRC) is a cluster of highly related species, both morphologically and phylogenetically [1]. The remaining data sets (3–6; Table 1) pertain to distinct species.

Distribution data was obtained from location points with a spatial accuracy greater than or equal to one square kilometer [1]. Sampling was performed across the Iberian Peninsula during the spring and summer seasons from 2013 to 2017, with random fieldwork points being selected near roads crossing forests and meadows at various altitudes, often in sparsely human-populated areas. Specimens were manually collected into a vial.

### 2.3. Habitat Suitability/Distribution Model Development

Species Distribution Models (SDMs) were developed in R statistical software [18] using the biomod2 package [15,16]. This package implements a multi-model ensemble forecasting approach that combines several statistical and machine-learning-based algorithms, thus enabling to assess and preventing a range of methodological uncertainties in each modelling algorithm and examining species-environment relationships. Models were fitted using ten modelling techniques: GLM (Generalized Linear Models); GBM (Generalized Boosted Models); GAM (Generalized Additive Models); CTA (Classification Tree Analysis); ANN (Artificial Neural Networks); FDA (Flexible Discriminant Analysis); MARS (Multivariate Adaptive Regression Splines); RF (Random Forests); MAXENT.Phillips and MAXENT.Tsuruoka (both Maximum Entropy-based models), currently available in biomod2. Default parameters were employed (except for the smoothing degree term in GAM, which was set to k = 4 to avoid over-fitting issues [19], the number of boosting trees in GBM, n.trees = 2000 and, the calculation of ‘hinge’ and threshold’ features both equal to FALSE for MAXENT. Phillips.

### 2.4. Environmental Variables Selection

Environmental variables (or predictors) were selected based on previous knowledge about the target species describing their general ecological preferences [1]. These included data at several scales from coarser climate variables related to temperature and precipitation regime and finer-grained variables including geomorphology, soil, and vegetation indices from satellite remote sensing (see Supplementary Materials, Appendix A).

A pre-selection of variables (see Supplementary Materials–Appendix A for the complete list) was performed in two stages to select the best performing predictors. First, a preliminary round of models, including all available species records (‘merged species dataset’ listed above) and all variables based solely on the Random Forest algorithm—known for its ability to handle large numbers of variables and multicollinearity in input data (i.e., by employing ‘feature bagging’ [20,21]—allowed selecting those variables with the highest predictive ability. A total of five pseudo-absence (PA) sets and 20 evaluation rounds were performed with a train/test partition of 70%/30%. Variable importance was calculated based on the biomod2 internal method, which calculates 1—Pearson’s correlation between reference predictions and predictions for a ‘randomized’ version of each variable. The highest the score, the greater is the influence of a variable in model predictions. A value of zero assumes no influence of a given variable. These variable importance scores were averaged across all PA sets, train rounds, and algorithms and were used to rank predictors.

Second, an iterative selection of variables was performed to decrease multicollinearity and increase parsimony based on the importance rank. This procedure was accomplished by testing the pairwise non-parametric Spearman correlation between each variable pair, starting from the top-most important to the least ranked. If a variable obtains all correlation values lower than 0.7, then it would be inserted in the final set. A total of six “best” variables were included in this parsimonious set and used for developing the final models (Table 2).

### 2.5. Model Fitting and Evaluation

Given that only presence data was available for the selected species, we obtained a total of five sets of randomly generated pseudo-absences, each with ten times the number of presence records. Since no previous information was assumed about species prevalence (p), model weights were adjusted to set *p* = 0.5 (biomod2 default), giving similar weight to presences and generating pseudo-absences.

Holdout cross-validation was employed to evaluate the models, with 80% of the input records used for model fitting and 20% for model evaluation at each round. A total of ten rounds were performed for model evaluation. For assessing model performance, the Area Under the Receiver-Operating Curve (AUC), the True-skill Statistic (TSS), and the Sensitivity and the Specificity values were calculated [16].

From biomod2, we calculated variable importance scores (following the same procedure as before in variable selection) for the final set of selected variables (Table 2), allowing us to rank each predictor relevance for modelling habitat suitability. Response curves, following the method described by Elith et al. [22] method, were used to plot how each variable influences habitat suitability values and obtain greater insight into each species environmental requirements.

### 2.6. Model Ensembles

Given that 500 models were generated per species, the less performant models were filtered out before the final ensemble forecasting. Hence, we selected the top 10% percentile best models for the seven best-performing techniques considering the TSS rank (n = 35). Based on these top-performing models, an ensemble using the average was implemented, thus reducing inter-model uncertainty. The threshold value maximizing the TSS statistic was used to ‘binarize’ projections (to dichotomous suitable/unsuitable habitat).

### 2.7. Habitat Similarity and Clustering Analysis

We performed a hierarchical clustering based on habitat suitability modeled projections from previous steps to assess the target species’ similarities in modeled habitat suitability and their potential co-distribution patterns. A pairwise distance matrix (d) was calculated as di,j = 1 − Spearman’s correlation, with i and j equal to two different ant-like fly species. The average linkage method was implemented to obtain the dendrogram in R software.

*Tachydromia* distribution overlap with oak (*Quercus*) species and biogeographic Life zones.

We hypothesize that the Iberian ant-flies distribution is deeply related to their ecological requirements, which seem to include (among other aspects) the leaf litter conditions where they inhabit. Moreover, their spatial distribution seems to be broadly related to different oak forests [1]. As such, we devised a spatial analysis to evaluate the co-distribution of ant-like flies with each type of Iberian oak forest and to address similarities in bioclimatic and biogeographic features.

We used Life zones to implement these analyses, which express the relationship between potential natural vegetation and bioclimatic drivers. These zones predominantly correspond to the spatial range of oak forests in the Iberian Peninsula as maximum representatives of each edaphic and climatic niche [10,23,24,25]. Life zones encompass major biogeographic sectorization of the Iberian Peninsula [10,26] including the Eurosiberian region, with a predominant temperate macrobioclimate, that encompasses the potential distribution of the fully deciduous oaks from Section *Quercus* (*Q. orocantabrica*, *Q. petraea*, and *Q. robur*) [27]. In contrast, the submediterranean variant of the temperate macrobioclimate corresponds to areas commonly fed by moderate summer precipitation, ascribed to the potential distribution of marcescent oaks (*Q. broteroi, Q. canariensis, Q. xcerrioides, Q. faginea, Q. lusitanica, Q. marianica, Q. pyrenaica,* and *Q. subpyrenaica*) [9,28], with the overlap areas between both, considered as transitional between temperate to submediterranean. By exclusion, the Mediterranean areas are mainly linked with the potential distribution of evergreen oaks (*Q. coccifera*, *Q. suber*, and *Q. rotundifolia*) from subgenus *Cerris* [8,28].

This relationship between oak species distribution and biogeographic regionalization was established through HSM’s, and the distribution maps for each *Quercus* species were obtained from [29]. A total of 13 *Quercus* species representative of Iberian deciduous and marcescent oaks from Section *Quercus* [28] were evaluated. The true-to-type Mediterranean life zone was promptly mapped by excluding the half of Iberia, not intersected with temperate and submediterranean oaks potential distribution.

For assessing the spatial overlap and unveiling potential co-distribution patterns between target *Tachydromia* species and oak forests (by dominant species) as well as Life zones, we calculated the intersection over union (***IoU***) index (Equation (1)).

(1) $$\mathrm{IoU}=\frac{A_{\mathrm{Tachydromia\_sp}}\;\cap \;A_{\mathrm{Quercus\_sp}}}{A_{\mathrm{Tachydromia\_sp}\;}\;\cup \;A_{\mathrm{Quercus\_sp}}}$$

In Equation (1), $A_{\mathrm{Tachydromia\_sp}}$ equals the potential distribution area of each ant-like fly species and, $A_{\mathrm{Quercus\_sp}}$ equal to the potential distribution of each *Quercus* species (or Life Zone). In Equation (1), the numerator equals the spatial intersection between distributions and the denominator to the spatial union. This calculation was performed for each pair of species and Life zones and approximates the value ***IoU*** = 1 when the similarity between co-distributions or overlap is higher.

## 3. Results

### 3.1. Predictive Model Performance

Overall, model evaluation scores (Table 1) show that high predictive performances were obtained for the ensemble model, with TSS values greater than 0.85 (ranging from −1 to 1, with the latter indicating perfect prediction), showing a good match between observed and predicted occurrences. Specifically, the highest TSS values were above 0.95 for *Tachydromia lusitanica*, *T. semiaptera*, and *T. iberica*. High predictive values were also obtained for ROC (ranging from 0 to 1), with values greater than 0.95 for all species and data aggregations, while KAPPA (ranging from −1 to 1) model accuracy scores were all equal or above 0.55.

Sensitivity and specificity represent the percentage of presences and absences correctly predicted by the model, respectively. In correspondence to the first scores, high values were also obtained for both metrics for all sets of data (i.e., both combined and individual records (Table 3), with the single-species datasets generally obtaining the highest values (sensitivity = 100; specificity ≥ 95). The MSD dataset obtained the lowest values (sensitivity = 89.9; specificity = 88.9), followed by the PRC dataset (sensitivity = 96.4; specificity = 90.1).

### 3.2. Relative Importance of Environmental Variables

**Figure 2:**
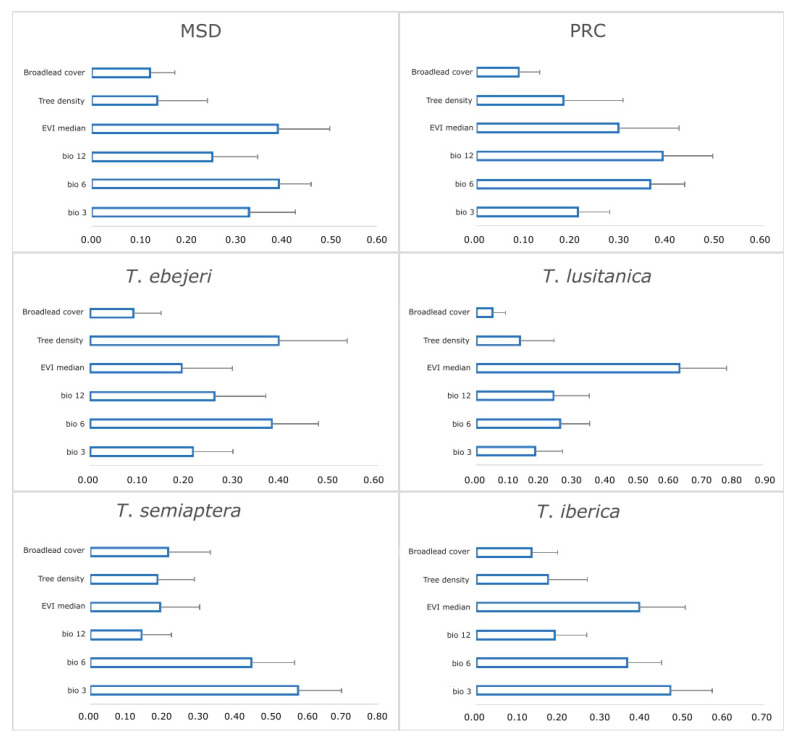
The relative importance of the abiotic and biotic variables used to predict the distributions of the individual species and groups thereof (i.e., MSD—merged species dataset with all species pooled together and PRC—most-phylogenetically related species). Bars indicate the mean value of relative importance obtained from the ten different modelling algorithms, while the whisker indicates the ½ standard deviation. EVI—Enhanced Vegetation Index; bio 3—isothermality; bio 6—Minimum Temperature of Coldest Month; bio 12—Annual Precipitation.

Variable importance scores were obtained by averaging values across all models, allowing to rank the predictive ability of each variable. Overall, results show large standard deviation values for all factors, which indicates a considerable variability regarding the influence of each predictor variable on each species, implying differences across modelling algorithms. For the MSD, the temperature-related variables and the EVI median are the most influential factors (Figure 2). For the PRC, the most influential is the annual precipitation, the minimum temperature of the coldest month, and, to a less extent, the EVI. For *T. ebejeri*, the most influential factors are tree density and the minimum temperature of the coldest month. For *T. lusitanica*, the EVI is the most crucial factor, with the minimum temperature of the coldest month and annual precipitation having similar but smaller importance. For *T. semiaptera*, isothermality and the minimum temperature of the coldest month are the most important factors, with all the remaining variables having considerably less and similar weight. Finally, *T. iberica* seems most influenced by isothermality, EVI, and the coldest month’s minimum temperature.

### 3.3. Species Hierarchical Clustering

**Figure 3:**
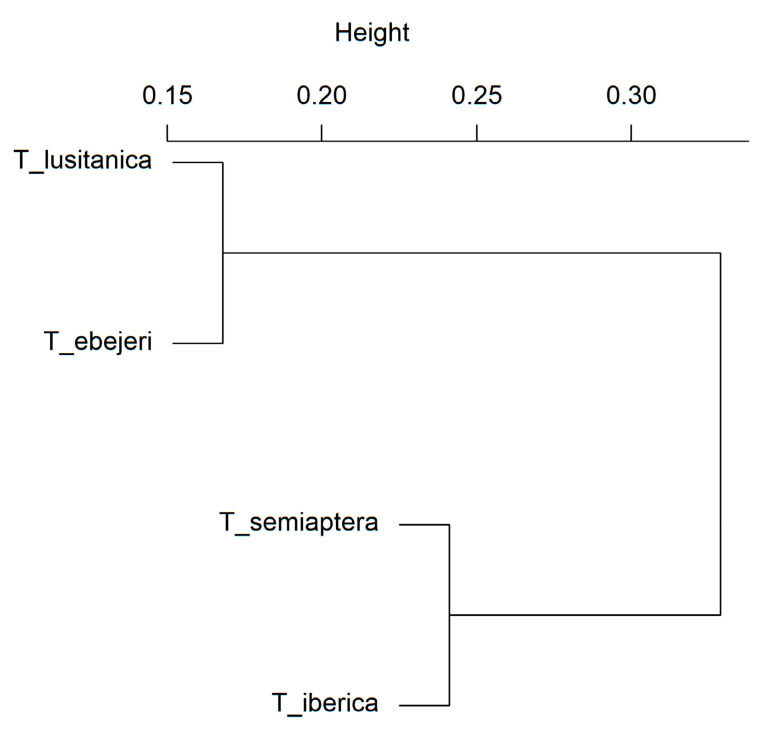
Hierarchical clustering dendrogram based on habitat suitability. Distance matrices were calculated as d = 1 − Spearman’s correlation. The average linkage method was implemented.

Two main clusters were formed, one consisting of *T. lusitanica* and *T. ebejeri* and the other comprising *T. semiaptera* and *T. iberica*, the latter showing a stronger similarity in habitat suitability and distribution (Figure 3).

### 3.4. Environmental Suitability

#### 3.4.1. Abiotic Factors

**Figure A1:**
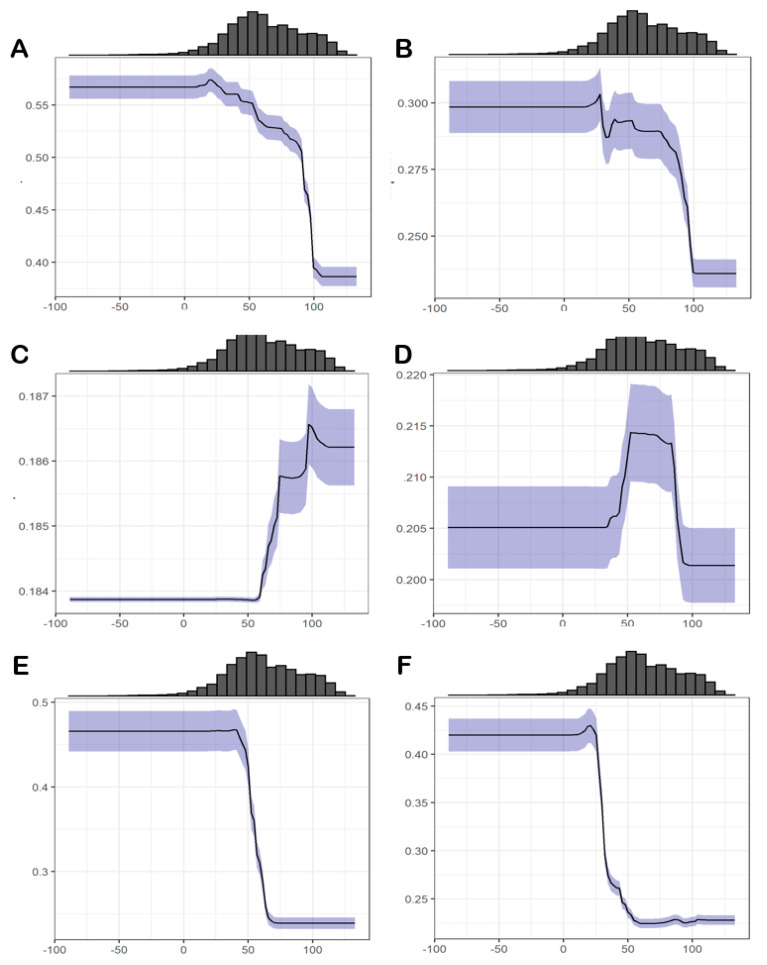
Response curves (average of all GBM models ± ½ std.-deviation) showing habitat suitability variation as function of isothermality values (bio3), for (**A**) MSD, (**B**) PRC, (**C**) *T. lusitanica*, (**D**) *T. ebejeri*, (**E**) *T. semiaptera* and (**F**) *T. iberica*.

Response curves allow obtaining detailed plots of the influence of each variable in habitat suitability values. Results indicate that most species are better adapted to relatively small temperature fluctuations (i.e., low isothermality; bio 3—min: 28%, average: 40%, max: 49%, see Supplementary Materials–Appendix B, Figure A1), with habitat suitability sharply decreasing around values of 35–40% of thermal oscillation within a month relative to the year. However, *T. lusitanica* shows a contrasting response, as its habitat suitability increases above the value of 40%.

**Figure A2:**
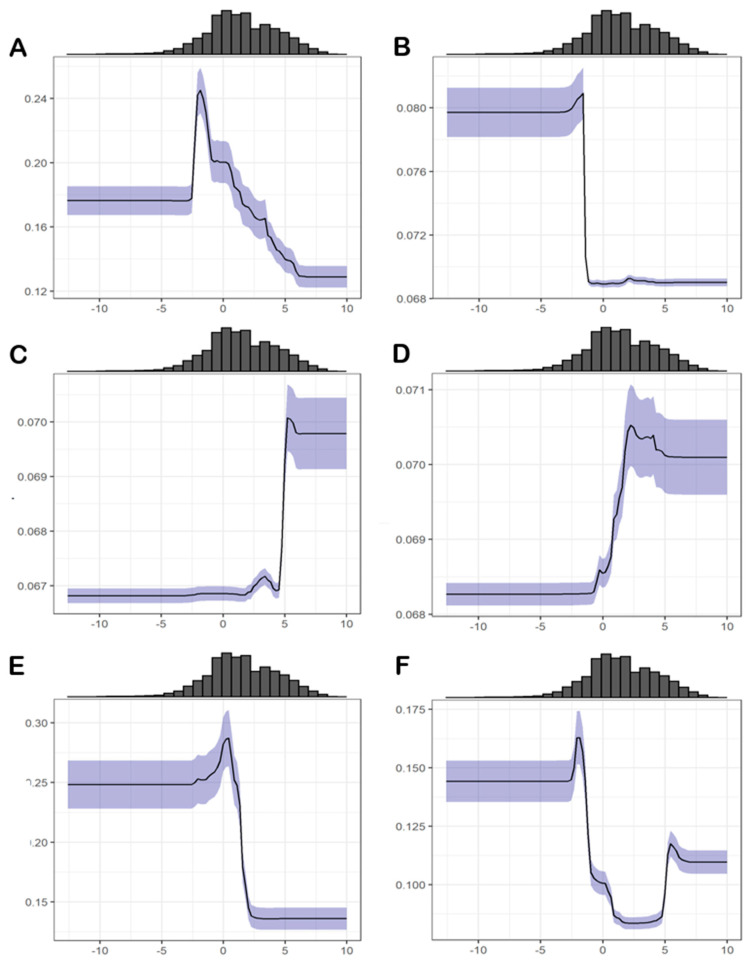
Response curves (average of all GBM models ± ½ std.-deviation) showing habitat suitability variation as function of the temperature of the coldest month (bio 6), for (**A**) MSD, (**B**) PRC, (**C**) *T*. *lusitanica*, (**D**) *T*. *ebejeri*, (**E**) *T*. *semiaptera* and (**F**) *T*. *iberica*.

Regarding the response to the minimum temperature of the coldest month (bio 6—min: −13 °C, average: 2 °C, max: 11 °C; see Supplementary Materials–Appendix B, Figure A2), habitat suitability of MSD and PRC sharply decreases in areas where temperatures reach near 0 °C, with an optimal response between -2.5 and 0 °C in the case of the first group. Species *T. lusitanica* and *T. ebejeri* are better adapted to temperatures above 0 °C, with an optimal response above 5 °C in the first and above 0 °C in the latter. *T. semiaptera* and *T. iberica* oppositely respond to this variable, as both seem to be better adapted to temperatures around or below 0 °C and then a sharp decrease in suitability is observed. The optimal response is between −2.5 °C and 2.5 °C for *T. semiaptera*, and between −2.5 °C and 0 °C for *T. iberica*.

**Figure 4:**
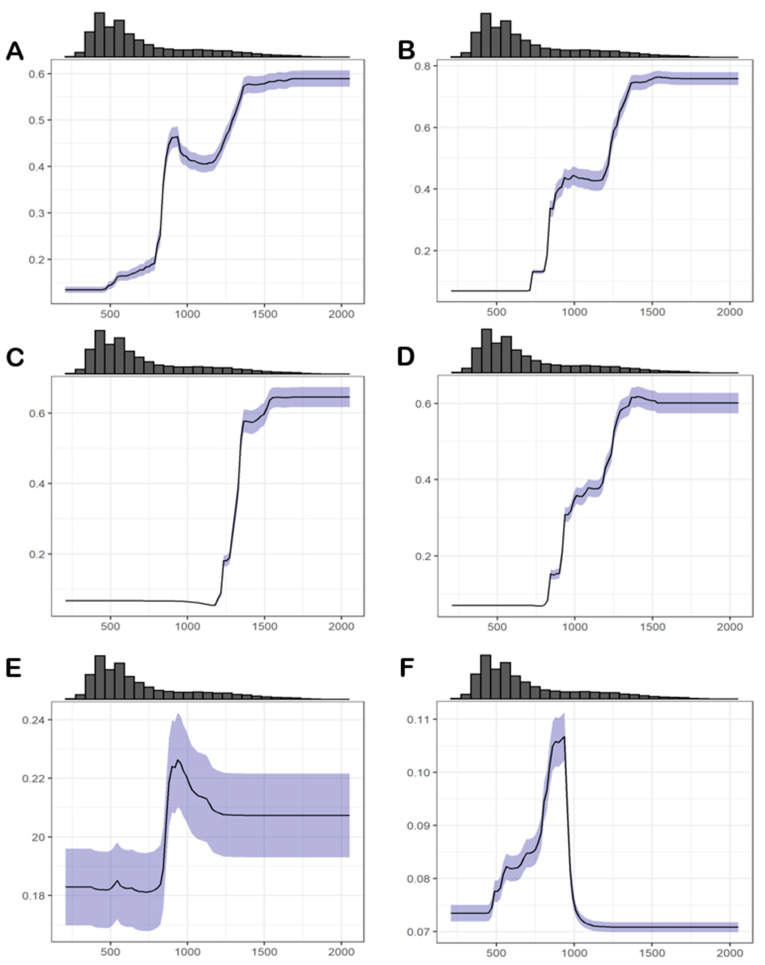
Response curves (average of all GBM models ± ½ std.-deviation) showing habitat suitability variation as a function of the annual precipitation values (bio12; in mm/year) for (**A**) MSD, (**B**) PRC, (**C**) *T. lusitanica*, (**D**) *T. ebejeri*, (**E**) *T. semiaptera* and (**F**) *T. iberica*.

The response curve concerning annual precipitation (Figure 4; bio 12—min: 208 mm, average: 698 mm, max: 2055 mm) indicates that for the MSD, PRC, and *T. ebejeri*, habitat suitability generally increases with the increment in precipitation from around 500 mm up to 1500 mm. *T. lusitanica* habitat relates to higher precipitation values than the remaining species (higher than 1000–1500 mm). The habitat suitability for *T. semiaptera*, on the other hand, increases up to around 1000 mm, then decreasing. *T. iberica* seems to require the least amount of precipitation among species, with habitat suitability values increasing from around 500 mm to nearly 1000 mm, then sharply decreasing.

#### 3.4.2. Biotic Factors

**Figure A3:**
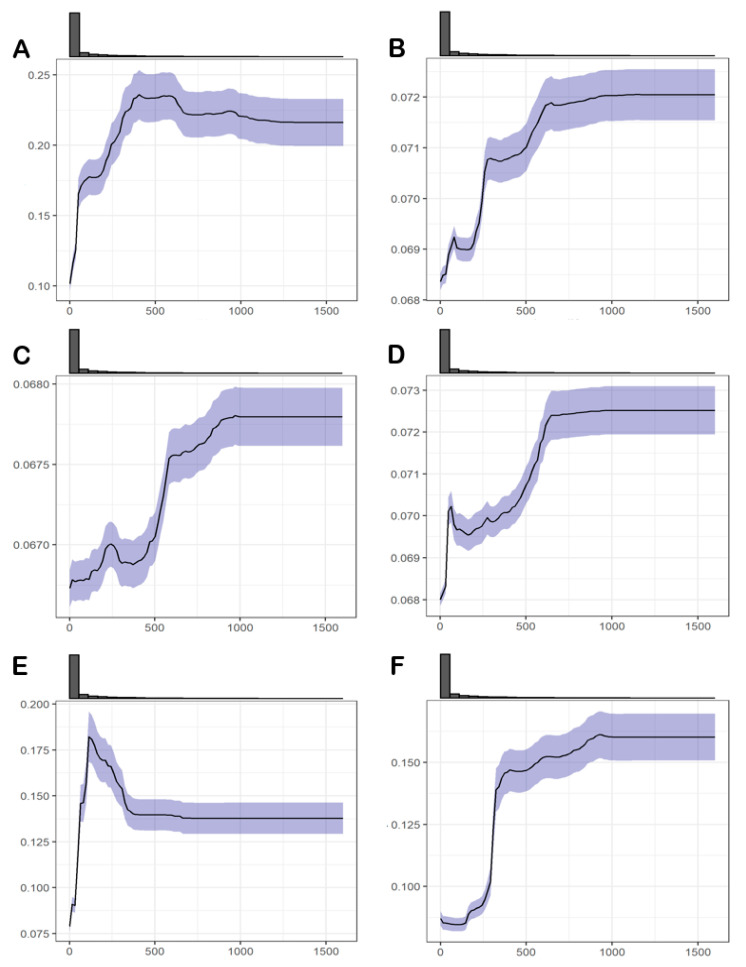
Response curves (average of all GBM models ± ½ std.-deviation) showing habitat suitability variation as function of broadleaf cover, for (**A**) MSD, (**B**) PRC, (**C**) *T*. *lusitanica*, (**D**) *T*. *ebejeri*, (**E**) *T*. *semiaptera* and (**F**) *T*. *iberica*.

**Figure A4:**
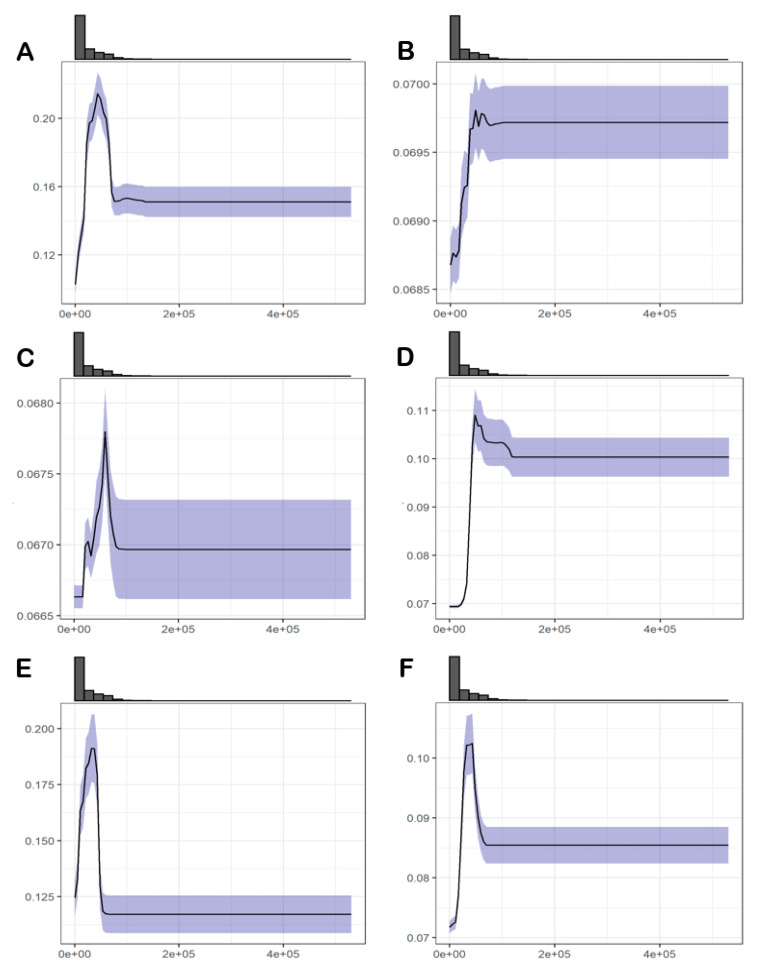
Response curves (average of all GBM models ± ½ std.-deviation) showing habitat suitability variation as function of tree density, for (**A**) MSD, (**B**) PRC, (**C**) *T. lusitanica*, (**D**) *T. ebejeri*, (**E**) *T. semiaptera* and (**F**) *T. iberica*.

Overall, the habitat suitability of most species shows a clear positive response to the increment of broadleaf forest cover (min: 0%, average: 8%, max: 100%; see Supplementary Materials—Appendix B, Figure A3). Nevertheless, *T. semiaptera* seems better adapted to habitats with a lower broadleaf forest cover than the other species. *T. semiaptera* is also adapted to habitats with an overall lower density of trees (min: 0, average: 21,058, max: 530,750 trees/km^2^) than the remaining species (see Supplementary Materials—Appendix B, Figure A4) (response peak from approx. 0 to 48,000 trees/km^2^). On the other hand, *T. ebejeri* is adapted to the highest tree density (response peak from approx. 42,000 to 112,000 trees/km^2^) of all species. *T. iberica* is present in habitats with tree densities (number of trees/km^2^) ranging from approx. 21,000 to 64,000. *T. lusitanica* is better adapted to habitats with slightly higher tree densities, from approx. 37,000 to 80,000. Species clusters, both MSD and PRC, have similar values of peak habitat suitability response to tree density, ranging from 37,000 to 75,000 (PRC) and 25,000 to 60,000 (MSD). As for EVI (min: −0.2, average: 0.17, max: 0.6), values around 0.3 were related to highly suitable locations for the occurrence of all species, linking them to areas with moderate to high greenness and biomass.

### 3.5. Potential Distribution Geographic Patterns

#### 3.5.1. Merged Species Dataset (MSD)

Overall, suitable habitats are primarily present in regions under a submediterranean and temperate bioclimatic influence, mainly in mountainous areas. Suitable habitats are mainly located in the N-NW Iberian Peninsula, except for central Galician Massif.

Moreover, large areas of predicted suitable habitat are present across the coastal Iberian System and in the Catalonian coastal range, including the Pyrenees. The Central System corresponds to the largest continuous area of suitable habitat. Unsuitable regions comprise most of the Southern parts of the Peninsula and, especially, the warmer and dryer plains (e.g., northern and southern sub-plateau and Ebro depression) with typical Mediterranean influence where evergreen forests have a higher representation [8,30].

#### 3.5.2. Phylogenetically Most Related Cluster (PRC)

Predicted suitable habitats for the PRC encompass a larger extent than individual model predictions for *T. lusitanica* and *T. ebejeri*. Thus, suitable habitats are expected to occur in eastern parts of the Central System and Sierra de Carzola and along the Catalonian coastal range.

- *Tachydromia lusitanica*

Suitable habitat range occurs most at the NW portion of the Iberian Peninsula and northern coastal Portugal, mainly under a temperate bioclimatic influence. Suitable habitats are also predicted to exist in disjunct areas in Serra da Lousã (Central System) and Serra de Sintra. Additionally, small habitat islands are located further away, in the Catalonian coastal range and the coastal Iberian System. Other suitable habitat “islands” were also found in, e.g., Serra de Monchique and in the southern section of the Penibaetic System.

- *Tachydromia ebejeri*

Suitable habitats are predicted to occur in most of the NW-CW Iberian Peninsula, with a clear exception to the extreme NW of littoral Galicia. Small habitat “islands” are predicted to occur in Serra de Marvão (from where a population is already known), Serra de Sintra, Serra de Monchique, Los Alcornocales Natural Park in the Penibaetic System, and small areas in Sierra de Guadarrama (Central System). The presence in the Central System is predicted to be restricted to the Portuguese portion and Sierra de Gata in Spain. Suitable areas are also expected to exist along with the Catalonian coastal range.

- *Tachydromia semiaptera*

Suitable habitats for *T. semiaptera* are predicted to occur in several areas under submediterranean influence, such as the Spanish Central System, in a large continuous area located above the NW part of the Spanish Central System, Montes de Toledo, and, in smaller areas, at Sierra Madrona and the Penibaetic System.

- *Tachydromia iberica*

This species is expected to have suitable habitats in various disjunct locations under the temperate and submediterranean bioclimatic influence. Larger areas of continuous suitable habitat are in three main submediterranean regions: Lusitanian Basin, Central System (Sierra de Guadarrama, Sierra de Gredos, and Sierra de Gata, Central System, Spain), and the southern part of the Penibaetic System (Los Alcornocales Natural Park, Andalusia, Spain).

#### 3.5.3. Distribution Overlap of *Tachydromia* with *Quercus* Species and Life Zones

The highest values of distribution overlap between species and groupings of *Tachydromia* with *Quercus* species (Table 4) are with *Quercus pyrenaica* and *Quercus robur* (>>0.50). *T. semiaptera* has the highest value of distribution overlap with a single species of oak, specifically with *Q. pyrenaica* (1), while also intersecting with *Q. robur* (0.41) and with *Quercus faginea* (0.36). The distribution of *T. lusitanica* overlaps with *Q. robur* (0.96) and *Q. pyrenaica* (0.64), having a much higher overlap with the first species. Similarly, *T. ebejeri* also mainly overlaps with *Q. robur* (0.93) and *Q. pyrenaica* (0.85). The distribution of *T. iberica* overlaps with various oak species, with the highest values associated with *Q. pyrenaica* (0.61) and *Quercus petraea* (0.50). Regarding the MSD, its distribution overlaps with several species, with the highest values for *Q. pyrenaica* (0.62) and *Q. robur* (0.54). Finally, the distribution of PRC mainly coincides with that of *Q. pyrenaica* (0.68) and *Q. robur* (0.77).

The distribution of the studied *Tachydromia* intersects predominantly with the Temperate Eurosiberian life zone (Table 5). The highest value of distribution value is found in *T. lusitanica* (0.82), followed by *T. ebejeri* (0.75), MSD (0.64), and PRC (0.69). The distribution of *T. semiaptera* and *T. iberica* also overlaps with the mentioned life zone (0.37 and 0.36, respectively). However, the first species overlaps more with the Eurosiberian Submediterranean Transition (0.47) and the latter with the Submediterranean (0.43).

## 4. Discussion

### 4.1. Multi-Scale Influence of Environmental Variables on Habitat Suitability

Our ensemble modelling approach investigated the potential distribution and the multi-scale environmental drivers shaping the ant-like flies’ habitat suitability. Model-based projections were also used to uncover the connection between their distribution and that of the different types of Iberian oak forests, allowing to provide guidelines for these flies’ conservation and future prospection.

Overall, the most important predictors of habitat suitability vary according to species and species clusters. However, our results support that, variables linked to temperature and precipitation—especially the minimum temperature of the coldest month and annual precipitation—have the highest predictive importance. These are followed in importance by variables related to vegetation greenness/biomass as captured by satellite-based EVI and, in the single case of *T. ebejeri*, tree density.

Therefore, both abiotic (climatic variables) and biotic factors (forest type and structure) play an important and interconnected role in explaining most of these species’ occurrences. These roles are expressed at different scales of variation (Vicente et al., 2014) with coarse (regional) factors, mainly linked to climate, explaining most of the habitat suitability and potential distribution macro-patterns followed by fine (local) scale drivers, mainly linked to forest type and structure, depicting actual habitat availability.

### 4.2. The Influence of Abiotic (Coarse-Scale) Variables

Temperature is generally known to regulate the metabolism, physiology of insects and to affect interactions between insects and plants [31,32], while precipitation has a crucial role in ecosystems, especially by influencing soil moisture [33]. In the case of the ant-like flies, it is expected that the importance of the minimum temperature of the coldest month may result from its critical role in regulating the emergence of adults, as they tend to become active in the very early spring in most of their distribution.

Annual precipitation probably acts as a proxy for the importance of soil humidity for ant-like flies, but the latter being much harder to estimate widely [34]. Generally, the habitat suitability of all modeled ant-like fly species increases along with annual precipitation and with highly suitable locations showing values above average in the Iberian context. Leaf litter decomposition rates increase with edaphic humidity [35], a well-known fundamental ecological resource for soil-dwelling saprophyte organisms such as the Sciaridae larvae, on which the ant-like flies mainly feed on.

Regarding the role of temperature, it is important to note that tolerance to temperature oscillations is critical as stark variations can affect insect eggs’ viability and further development [36]. These stages are completed in the soil, under the leaf litter, for ant-like flies during the most extreme summer and winter temperatures and cannot tolerate desiccation or freezing. Larvae and eggs are the most vulnerable stage of soil-dwelling insect development, with their survival being dependent on the water content in the soil [37]. Being mostly distributed in the submediterranean life zone, *T. semiaptera* and *T. iberica* can cope with a slight degree of temperature seasonality (i.e., low temperature in the winter, high in the summer), while particularly *T. lusitanica*, occurs in areas under a Eurosiberian Temperate-most climatic influence (Table 5), likely does not tolerate wide temperature ranges. Nevertheless, all stages of the ant-like flies are likely to be dependent on the humidity, temperature, and structure of the leaf litter itself for regulating their physiology, finding food, and even escaping from predators, as the adults hide under the leaf litter as a response to perceived approaching danger [1].

### 4.3. The Influence of Biotic (Fine-Scale) Factors

Biotic factors, represented here by forest structure and composition, determine habitat availability at a local scale for ant-like flies. In this sense, satellite-based vegetation indices such as EVI depict that these species inhabit areas with higher biomass and leaf area index than the Iberian Peninsula average. This is also reflected in these species’ occurrence in forested habitats, particularly those composed of broadleaf trees, such as oaks and beech [1]. This connection is apparent when analyzing the overlap of predicted suitable distribution of ant-like flies and oak forests in the Iberian Peninsula.

*Tachydromiasemiaptera* preference for broadleaf forests with less tree density may be related to the typical structural features of the woodlands where it tends to occur at a finer scale. Due to its transitional character, *Q. pyrenaica* forests found at the southern limit of their range exhibit highly variable tree densities, mainly depending on the intensity of human intervention (e.g., timber production) [38].

Another important factor driving this preference may be related to the phenology of *T. semiaptera*, in which adults tend to become active later in the year than other ant-like species (late spring) at low altitudes. At a micro-scale, all ant-like flies seem to be preferentially active in areas where the leaf litter is concentrated while directly illuminated by sunlight [1]. During early spring, the canopy of marcescent and deciduous oak forests lacks dense foliage, allowing sunlight to reach the ground. In such conditions, the ant-like flies likely benefit from the warmer temperatures while still protected among the leaf litter from predators, where they can also find their prey. Inhabiting in forests of broadleaved oaks such as *Q. robur* and *Q. pyrenaica* in the Eurosiberian Temperate life zone, this scenario is typically experienced by *T. ebejeri*—which shows a preference for the highest tree density—and by *T. lusitanica*. Since, in late spring, the leaves of dense woodlands block the sunlight from reaching the ground, *T. semiaptera* may prefer more open areas or even edges of denser stands.

This pattern was observed (op. cit.) in ant-like fly species in the summer at high altitudes, where *Fagus sylvatica* substitutes oak forests. This species often forms very dense woodlands with a fully covered canopy that does not allow sunlight to reach the ground [39]. In these cases, *T. pandellei*, *T. pieltaini,* and *T. apterygon* (a related non-Iberian species) were only found at the edge of the forest or in clearings, with abundant leaf litter and low herbaceous vegetation, being apparently absent from nemoral forest environments and surrounding meadows.

### 4.4. Spatial Overlap between Ant-like Flies, Oak Forests, and Life Zones in the Iberian Peninsula

Connecting species distribution to well-defined Life zones is essential to understand the different sets of biotic and abiotic factors shaping their distribution, and, thus, it can be a very useful tool for conservation. In the specific case of the ant-like flies, the oak forests’ distribution seems to be an important predictor of their occurrence, being distributed according to the same Life zones and intrinsic macrobioclimatic drivers. Comparing these three elements allows obtaining a better notion of where the ant-like flies may occur at a finer scale.

Therefore, *T. semiaptera* is a species connected with the well-defined submediterranean life zone, as reflected by the complete overlap between its distribution and *Q. pyrenaica*. On the other hand, *T. iberica* appears to be well adapted to this life zone but also shows likely adaptability to the submediterraneanTemperate Transition zone, as suggested by the distribution overlap with *Q. pyrenaica* and *Q. petraea* [29], also evidencing affinities with more continental areas, subdued to higher temperature variations/seasonality.

*Tachydromia iberica* seems to have a wider potential distribution than *T. semiaptera*. However, it is also clearly absent from the Temperate Eurosiberian life zone, where *T. lusitanica* and/or *T. ebejeri* are expected to occur, which is most evident in the Catalonian coastal range and the Galician massif. Additionally, its presence is very likely at the Los Alcornocales National Park area, which receives horizontal precipitation in the summer. Indeed, there are several habitat patches that retain high humidity in the submediterranean life zone and, thus, function as ecological “islands” [8,9]. Due to their inherent Temperate Eurosiberian influence, these patches act as refugia for paleoclimatic relict forests. These can include broad marcescent forests dominated by *Q. pyrenaica*, *Q. faginea,* and *Q. canariensis* [29,40,41] relevant for ant-like flies’ conservation.

**Figure 5:**
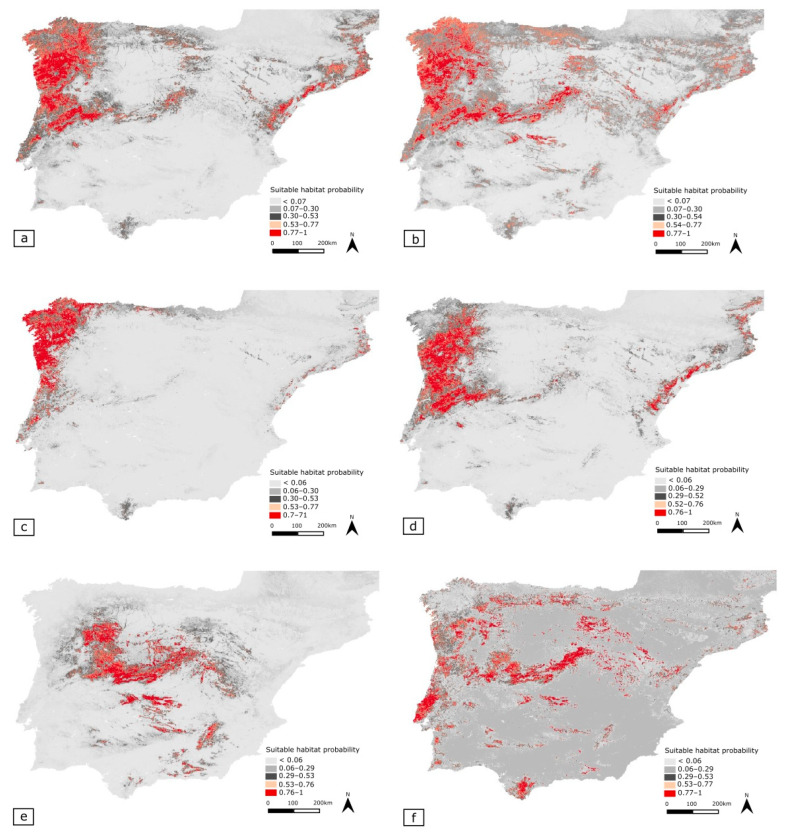
Maps representing the predicted habitat suitability, generated from the ensemble of ten models implemented on biomod 2 workflow. Each map represents either one of the two approaches for developing models through pooled (**a**,**b**) or individual species records (**c**–**f**). These sets are: (**a**) Merged Species Dataset including all nine species records (MSD), (**b**) most phylogenetically related cluster (PRC) of species, (**c**) *Tachydromia lusitanica*, (**d**) T. *ebejeri*, (**e**) *T. semiaptera* and (**f**) *T. iberica*. Colour represent habitat suitability value, with light grey corresponding to low suitability, red with high suitability.

*Tachydromia lusitanica* is a full Temperate-Eurosiberian species with the highest requirements concerning annual precipitation and absence of winter cold (Figure 4), occupying the coastal areas where this life zone is most significant. The potential distribution of *T. lusitanica* is entirely adherent to the extreme littoral shore of the NW Iberian Peninsula, overlapping almost exclusively to the potential distribution of *Q. robur*, a dominant species of the climax Temperate Eurosiberian forests at low altitude (Figure 5, Table 4 and Table 5). Its potential distribution is also apparent in the coastal areas at medium to high altitudes in dry Mediterranean regions, such as Serra de Sintra, Serra de Monchique, and Catalonian coastal range, fed by moderate horizontal precipitation summer. This phenomenon can be originated either by advection fogs from the sea or from cyclogenetic processes in the Mediterranean Sea that lead to heavy rains [29,41,42,43,44,45]. Consequently, these types of ecosystems can very well support this species’ habitat requirements.

The predicted high likelihood of *T. lusitanica* being the only ant-like fly inhabiting the Galician massif according to its adaptation to habitats with sharper Temperate Eurosiberian influence [46]. *T. ebejeri* tolerates higher temperature seasonality and minor summer drought, with its potential distribution greatly overlapping with that of *Q. pyrenaica* (Table 4), and its potential presence in the Catalonian coastal range, which deserves further prospection. Otherwise, it is expected that both species co-occur in much of their predicted distribution.

### 4.5. Conservation and Future Study Guidelines

Correctly assessing the conservation status of insects is not a straightforward approach, and it is known that the IUCN criteria—the most widely used tool—has various shortfalls in the case of invertebrates [47]. Even so, following IUCN guidelines, the conservation status of *Tachydromia lusitanica* in mainland Portugal has been assessed as Endangered (in press).

The reality is that the ant-like *Tachydromia* species are still poorly known and studied. As such, this research expanded the current knowledge, devised spatial predictions to improve sampling schemes, and planned fieldwork in areas that still need to be thoroughly surveyed. In this regard, it is essential to note that despite the model not retrieving geographical barriers (e.g., mountains and large rivers), which the ant-like flies may or may not be able to cross, it can spatially depict their realized ecological niche, helping to predict species occurrence and potential areas for new expeditions.

Such information will be beneficial to assess the global conservation status of the ant-like flies more reliably. The regions that need the most to be surveyed are mainly located in the southernmost part of the Iberian Peninsula, an area which may even likely contain undescribed species restricted to “habitat islands”. These areas include Monchique and surrounding mountains in southwest Portugal, the Penibaetic system, and the Catalonian coastal range. These particular forest-habitats, distributed across submediterranean ecotone-areas, which are mostly composed of marcescent oaks, will be severely affected by predictable climatic changes [8,9,29,39,48]. Particularly, the Iberian southwest and the Catalonian coastal mountains harbor remarkable oak taxa, including endangered species (e.g., *Quercus canariensis*) and species with unique evolutionary characteristics (e.g., *Q. estremadurensis* or *Q. xcerrioides* [49]). Hence, in these areas, both the genetic diversity of oak taxa and of the highly sedentary ant-like flies was shaped by the same phylogeographic conditions. Consequently, these regions encompass umbrella-habitats that commonly harbor highly specific biodiversity [38,50,51,52,53,54]. Additionally, it is fundamental to complete the knowledge gaps regarding the species already known with very few presence records: *T. stenoptera*, *T. cantabrica*, *T. nigrohirta*, *T. pieltaini,* and *T. pandellei*. It is also relevant to further understand the limits of the distribution of the ant-like flies in the Pyrenees, as *T. pandellei* is known from the French part of this range.

Furthermore, it would also be useful to gather more information about the life cycle of the ant-like flies with benefits for improving distribution models, specifically regarding the larval stage. Incorporating such knowledge into models, more specifically, physiological variables related to climate and phenology will potentially enhance the predictive power of models, helping to anticipate and manage future shifts in the biogeography of Iberian ant-like flies.

Ant-like flies face several threats due to global environmental changes. These are linked to climate change and habitat destruction, including urbanization, forest fires, land-use alterations, invasive species forming very thick stands, and changing the leaf litter composition and decomposition rate (e.g., *Acacia* spp.) [55]. It is important to note that vast areas of otherwise suitable habitat have been reclaimed for urbanization or agriculture. These threats are not reserved only to a single species but can apply to all ant-flies, with some particularities. For instance, in the Iberian south, climate change may be a particularly critical threat by rapidly turning the current “habitat islands” into unsuitable environments.

It is also noteworthy to point that, although the focal species may have a large distribution, the actual extent of occurrence locally tends to be limited to suitable pockets of micro-habitat. As such, due to these flies’ very low dispersal ability, all threats that fragment and reduce available habitat represent a severe risk, further eroding genetic variability and reducing their ability to evolve and cope with environmental change.

The conservation of the ant-like flies is highly dependent and connected to the very “health” of the oak and beech forests they inhabit. Hence, by preserving, recovering, and promoting the growth of native climax forests, we can ensure the survival of these flies and many other species typical of these highly diverse ecosystems. This can be achieved by limiting extensive tree falling for timber production, controlling woody invasive species such as those from the *Acacia* genus that are strongly present in the region [56,57], especially from areas where oaks are growing (often through natural regeneration), and planting oak trees where they are locally native.

### 4.6. Study Limitations and Future Improvements

Although best practices were implemented in model development, some limitations still apply to our approach. For instance, the MSD (i.e., set of data including the presence data from all species) aggregates the habitat requirements of ant-like flies that in this study we have observed to have substantial differences. In this case, this may have resulted in overfitting, which potentially hinders the ability to understand these flies’ general ecological and distribution patterns. In this case, improving the occurrence dataset in the future may help to overcome this limitation.

Furthermore, clustering phylogenetically related species may not be informative on itself if those species have evolved to adapt to highly distinct environments. Nevertheless, given the field observations and distribution (Figure 1), the species included in the PRC (*T. nigrohirta, T. stenoptera,* and *T. cantabrica*) seem to have similar habitat and climate requirements. This may have resulted in overestimated suitable areas compared to individual model predictions for *T. lusitanica* and *T. ebejeri*, but it does highlight the need to further prospect certain areas, as the Cantabrian range.

Finally, in future studies it would be important to collect biotic information regarding the microhabitat, such as leaf litter conditions (e.g., stage of biodegradation) and microclimatic data, so they can be included in habitat suitability modelling. Additionally, future studies will attempt to address variation in ecological conditions by implementing model projections based on climate and also land-use change scenarios (both past based on satellite time-series and future).

## 5. Conclusions

Our study provided crucial information and addressed a critical knowledge gap regarding the distribution of the Iberian ant-like flies, which inhabit different climatic and biotic envelopes. With the combination of climate and biotic variables, we showed that habitat suitability segregates two groups of species adapted to different climatic requirements, one group related to greater humidity and water availability and the other to drier and warmer conditions. The obtained maps are congruent with Iberian Biogeographic gradients and are useful to unveil crucial areas for conservation and new areas for the prospection of putative new and less widely distributed species. These areas are mainly located in southern Iberian submediterranean mountains, which function as “habitat islands”, allowing the co-occurrence of marcescent oak forests and ant-like flies. Overall, these oak forests act as very effective indicators for their presence. Finally, this work is of major importance to predict the effects of environmental change on the conservation of this taxonomic group of ant-like flies in the Iberian Peninsula and Southern Europe.

## Figures and Tables

**Table 1:** Sets of species data used in model development. Sets 1 and 2 used a pooling strategy where, respectively, all species or the most phylogenetically related ones were combined. For sets 3 to 6, each species data was used individually without pooling.

Dataset Number	Dataset Designation	Species Name and Total Number	Number of Presence Records
1	Merged species dataset (MSD)	*T. cantabrica* *T. ebejeri* *T. iberica* *T. lusitanica* *T. nigrohirta* *T. pandellei* *T. pieltaini* *T. semiaptera* *T. stenoptera* (9)	100
2	Cluster of most phylogenetically related species (PRC)	*T. cantabrica* *T. ebejeri* *T. lusitanica* *T. nigrohirta* (4)	53
3	*Tachydromia lusitanica*	(1)	20
4	*Tachydromia ebejeri*	(1)	20
5	*Tachydromia semiaptera*	(1)	14
6	*Tachydromia iberica*	(1)	20

**Table 2:** Selected variables used for developing habitat suitability models and respective data sources.

Type	Description	Source
Climate (coarse scale)	BIO 3—Isothermality (Mean Diurnal Range /Temperature Annual Range) (×100)	WorldClim v2.1 https://worldclim.org (accessed on 20 November 2021)
	BIO 6—Minimum Temperature of Coldest Month	
	BIO 12—Annual Precipitation	
Vegetation/land cover (fine scale)	% cover of broadleaf forests	Copernicus High Resolution Layers https://land.copernicus.eu/pan-european/high-resolution-layers (accessed on 20 November 2021)
	Tree density (nr/km^2^)	Crowther et al., 2016
	Enhanced Vegetation Index (EVI)—annual median	Terra/MODIS MOD13Q1 product (v6) https://lpdaac.usgs.gov/products/mod13q1v006/USGS/NASA (accessed on 20 November 2021)

**Table 3:** Ensemble model evaluation by Cohen’s Kappa, TSS and ROC model performance measures. MSD stands for merged species records and PRC for phylogenetically most related cluster. Sensitivity and Specificity are defined for the cut-off maximizing the TSS value (usually very similar to that of ROC).

	MSD	PRC	*T. ebejeri*	*T. lusitanica*	*T. semiaptera*	*T. iberica*
KAPPA	0.55	0.60	0.56	0.66	0.75	0.73
TSS	0.79	0.87	0.96	0.95	0.95	0.96
ROC	0.96	0.98	0.99	0.99	0.99	0.99
Sensitivity	89.9	96.4	100	100	100	100
Specificity	88.9	90.1	95.8	95.2	95.1	96.2

**Table 4:** Species distribution overlap: area intersected with *Quercus* species/total area target species. Oak species are: Qbro—*Q. broteroi*, Qcan—*Q. canariensis*, Qcer—*Q. xcerrioides*, Qest—*Q. estremadurensis*, Qfag—*Q. faginea*, Qlus—*Q. lusitanica*, Qmar—*Q. marianica*, Qoro—*Q. orocantabrica*, Qpet—*Q. petraea*, Qpub—*Q. pubescens*, Qpyr—*Q. pyrenaica*, Qrob—*Q. robur* and Qsub—*Q. subpyrenaica*.

	Qbro	Qcan	Qcer	Qest	Qfag	Qlus	Qmar	Qoro	Qpet	Qpub	Qpyr	Qrob	Qsub
MSD	0.03	0.02	0.00	0.03	0.10	0.03	0.04	0.09	0.37	0.21	0.62	0.54	0.16
*T. lusitanica*	0.00	0.00	0.00	0.00	0.02	0.03	0.00	0.00	0.06	0.00	0.64	0.96	0.00
*T. ebejeri*	0.00	0.00	0.00	0.00	0.09	0.00	0.00	0.01	0.09	0.01	0.85	0.93	0.00
*T. semiaptera*	0.00	0.00	0.00	0.01	0.36	0.00	0.00	0.00	0.00	0.00	1.00	0.41	0.00
*T. iberica*	0.14	0.08	0.00	0.14	0.29	0.09	0.13	0.14	0.50	0.34	0.61	0.06	0.33
PRC	0.00	0.00	0.00	0.01	0.12	0.03	0.00	0.05	0.19	0.06	0.68	0.77	0.03

**Table 5:** Species distribution overlap: area intersected with Life zones/total area target species.

	Mediterranean	Temperate Eurosiberian	Submediterranean	Eurosiberian Submediterranean Transition
MSD	0.07	0.64	0.16	0.13
*T. lusitanica*	0.04	0.82	0.00	0.14
*T. ebejeri*	0.03	0.75	0.03	0.19
*T. semiaptera*	0.00	0.37	0.17	0.47
*T. iberica*	0.08	0.36	0.43	0.12
PRC	0.08	0.69	0.06	0.17

**Figure A5:**
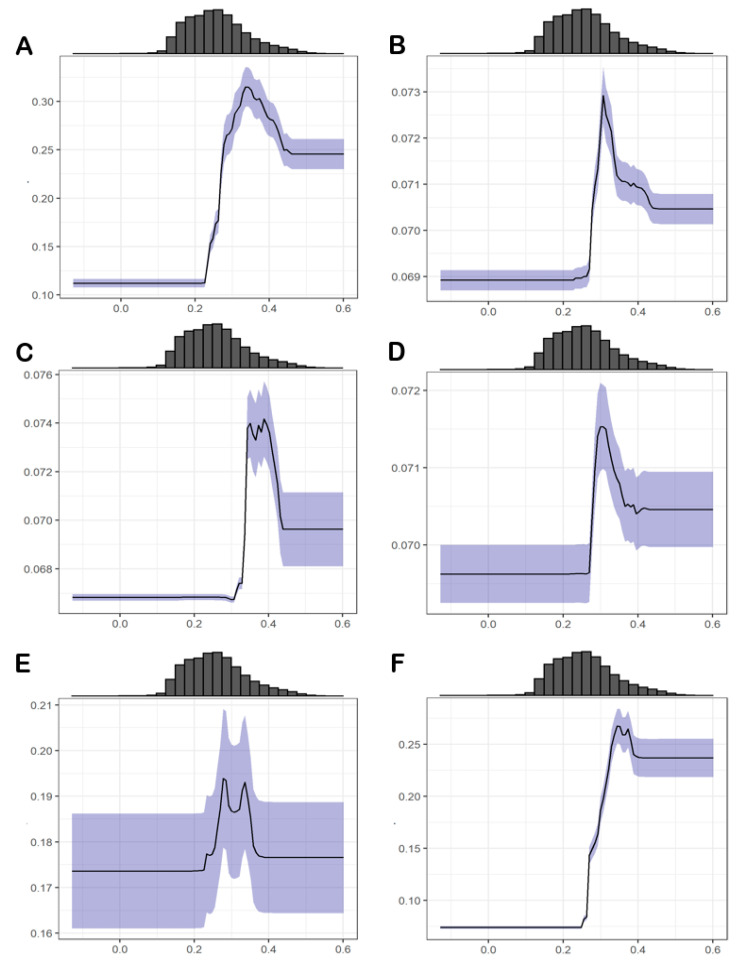
Response curves (average of all GBM models ± ½ std.-deviation) showing habitat suitability variation as function of EVI, for (**A**) MSD, (**B**) PRC, (**C**) *T*. *lusitanica*, (**D**) *T*. *ebejeri*, (**E**) *T*. *semiaptera* and (**F**) *T*. *iberica*.

## Data Availability

Upon acceptance of the manuscript, all set of data supporting results will be made publicly available at GitHub platform.

## Supplementary Materials

The following are available online at https://zenodo.org/record/5730507 (accessed on 20 November 2021).

## Appendix A

**Table A1:** Known environmental factors rationale based on previous expert-knowledge about the target species describing their broad ecological preferences, at different scales.

Scale	Main Environmental Factors Determining Habitat Suitability for the Target Species in Iberia	
Global	Climate	Atlantic
		Continental
		Submediterranean
Regional	Elevation	Mountainous regions
		Coastal areas
	Temperature	Mild in Spring and/or Summer
	Precipitation	Medium/high
Local	Soil	Leaflitter present
		High organic content
		High humidity
	Forest type	Deciduous
		Marcescent
	Tree cover	Partial to allow sunlight penetration

**Table A2:** Full list of variables and data sources.

Type	Variable Name	Data Sources
Climate	BIO1 = Annual Mean Temperature	WolrdClim bioclimatic indices v-2.1/https://www.worldclim.org/data/worldclim21.html. Accessed: 26 May 2020
Climate Geomorphology	BIO2 = Mean Diurnal Range (Mean of monthly (max temp–min temp))	WolrdClim bioclimatic indices v-2.1/https://www.worldclim.org/data/worldclim21.html. Accessed: 26 May 2020. SRTM 90 m Digital Elevation Database v4.1: https://cgiarcsi.community/data/srtm-90m-digital-elevation-database-v4-1/. Accessed: 26 May 2020
	BIO3 = Isothermality (BIO2/BIO7) (×100)	
	BIO4 = Temperature Seasonality (standard deviation ×100)	
	BIO5 = Max Temperature of Warmest Month	
	BIO6 = Min Temperature of Coldest Month	
	BIO7 = Temperature Annual Range (BIO5-BIO6)	
	BIO8 = Mean Temperature of Wettest Quarter	
	BIO9 = Mean Temperature of Driest Quarter	
	BIO10 = Mean Temperature of Warmest Quarter	
	BIO11 = Mean Temperature of Coldest Quarter	
	BIO12 = Annual Precipitation	
	BIO13 = Precipitation of Wettest Month	
	BIO14 = Precipitation of Driest Month	
	BIO15 = Precipitation Seasonality (Coefficient of Variation)	
	BIO16 = Precipitation of Wettest Quarter	
	BIO17 = Precipitation of Driest Quarter	
	BIO18 = Precipitation of Warmest Quarter	
	BIO19 = Precipitation of Coldest Quarter	
	Topographic Wetness Index (TWI)	
Soil attributes	% of sand	Topsoil physical properties for Europe (based on LUCAS topsoil data)/EU JRC: https://esdac.jrc.ec.europa.eu/content/topsoil-physical-properties-europe-based-lucas-topsoil-data. Accessed on 4 October 2018
Soil attributes	% of clay	Topsoil physical properties for Europe (based on LUCAS topsoil data)/EU JRC: https://esdac.jrc.ec.europa.eu/content/topsoil-physical-properties-europe-based-lucas-topsoil-data. Accessed on 4 October 2018. Soil pH in Europe/EU JRC: https://esdac.jrc.ec.europa.eu/content/soil-ph-europe. Accessed on 4 October 2018
	% of silt	
	% coarse fragments	
	Bulk Density	
	Available Water Capacity (AWC)	
	Soil pH	
	Topsoil organic matter content	European Soil Database & soil properties/EU JRC: https://esdac.jrc.ec.europa.eu/resource-type/european-soil-database-soil-properties. Accessed on 4 October 2018
	Root depth	European Soil Database & soil properties/EU JRC: https://esdac.jrc.ec.europa.eu/resource-type/european-soil-database-soil-properties. Accessed on 4 October 2018 Terra/MODIS product MOD13Q1 v-006/250m: https://lpdaac.usgs.gov/products/mod13q1v006/. Accessed on 4 October 2018
	Enhanced Vegetation Index (EVI)—annual median	
Remotely sensed vegetation attributes	Enhanced Vegetation Index (EVI)—annual interquartile range	Terra/MODIS product MOD13Q1 v-006/250m: https://lpdaac.usgs.gov/products/mod13q1v006/. Accessed on 4 October 2018 Global tree density map/Crowther, T. W., Glick, H. B., Covey, K. R., et al. (2015). Mapping tree density at a global scale. Nature, 525(7568), 201–205. doi:10.1038/nature14967. Available online: https://elischolar.library.yale.edu/yale_fes_data/1/. Accessed on 4 October 2018
	Global Tree Density/~1 Km	
	% cover of coniferous forest	Copernicus High Resolution Layers https://land.copernicus.eu/pan-european/high-resolution-layers. Accessed on 4 October 2018
	% cover of broadleaf forest	Copernicus High Resolution Layers https://land.copernicus.eu/pan-european/high-resolution-layers. Accessed on 4 October 2018 Terra/MODIS product MOD16A2 v-006/1000m: https://lpdaac.usgs.gov/products/mod16a2v006/. Accessed on 4 October 2018
	Evapotranspiration—annual median	
Remotely sensed water cycle attributes	Evapotranspiration—annual interquartile range	Terra/MODIS product MOD16A2 v-006/1000m: https://lpdaac.usgs.gov/products/mod16a2v006/. Accessed on 4 October 2018 Terra/MODIS product MOD09A1 v-006/500m: https://lpdaac.usgs.gov/products/mod09a1v006/. Accessed on 4 October 2018
	Normalized Difference Water Index (NDWI)—annual median	
	Normalized Difference Water Index (NDWI)—annual interquartile range	Terra/MODIS product MOD09A1 v-006/500m: https://lpdaac.usgs.gov/products/mod09a1v006/. Accessed on 4 October 2018
